# Identification of a Novel Eight-lncRNA Prognostic Signature for HBV-HCC and Analysis of Their Functions Based on Coexpression and ceRNA Networks

**DOI:** 10.1155/2020/8765461

**Published:** 2020-04-14

**Authors:** Xiaonan Zhao, Zhenzi Bai, Chenghua Li, Chuanlun Sheng, Hongyan Li

**Affiliations:** Infectious Department, The Third Hospital of Jilin University, Changchun 130033, China

## Abstract

Studies have demonstrated the prognosis potential of long noncoding RNAs (lncRNAs) for hepatocellular carcinoma (HCC), but specific lncRNAs for hepatitis B virus- (HBV-) related HCC have rarely been reported. This study was aimed at identifying a lncRNA prognostic signature for HBV-HCC and exploring their underlying functions. The sequencing dataset was collected from The Cancer Genome Atlas database as the training set, while the microarray dataset was obtained from the European Bioinformatics Institute database (E-TABM-36) as the validation set. Univariate and multivariate Cox regression analyses identified that eight lncRNAs (TSPEAR-AS1, LINC00511, LINC01136, MKLN1-AS, LINC00506, KRTAP5-AS1, ZNF252P-AS1, and THUMPD3-AS1) were significantly associated with overall survival (OS). These eight lncRNAs were used to construct a risk score model. The Kaplan-Meier survival curve results showed that this risk score can significantly differentiate the OS between the high-risk group and the low-risk group. Receiver operating characteristic curve analysis demonstrated that this risk score exhibited good prediction effectiveness (area under the curve (AUC) = 0.990 for the training set; AUC = 0.903 for the validation set). Furthermore, this lncRNA risk score was identified as an independent prognostic factor in the multivariate analysis after adjusting other clinical characteristics. The crucial coexpression (LINC00511-CABYR, THUMPD3-AS1-TRIP13, LINC01136-SFN, LINC00506-ANLN, and KRTAP5-AS1/TSPEAR-AS1/MKLN1-AS/ZNF252P-AS1-MC1R) or competing endogenous RNA (THUMPD3-AS1-hsa-miR-450a-TRIP13) interaction axes were identified to reveal the possible functions of lncRNAs. These genes were enriched into cell cycle-related biological processes or pathways. In conclusion, our study identified a novel eight-lncRNA prognosis signature for HBV-HCC patients and these lncRNAs may be potential therapeutic targets.

## 1. Introduction

Liver cancer, 90% of which is hepatocellular carcinoma (HCC), is one of the most common malignancies and the leading cause of cancer-related deaths worldwide [1, 2]. Despite great advances made in therapeutic approaches, relapse, progression, and metastasis rates remain high, which leads to poor prognostic outcomes [3]. Hepatitis B virus (HBV) infection is recognized as the most important risk factor associated with the biological aggressiveness of HCC and patients' survival [4]. Therefore, it may be an important issue to screen prognostic biomarkers and therapeutic targets for patients with HBV-HCC in order to optimize treatment, prevent progression, and improve the prognosis.

Recently, more and more evidence has highlighted the important roles of long noncoding RNAs (lncRNAs) in HBV-HCC via directly influencing the expression of their neighboring genes (coexpression hypothesis) or acting as microRNA (miRNAs) sponges to negatively regulate the expression of miRNA target genes (competing endogenous RNA (ceRNA) hypothesis) [5]. For example, Chen et al. found that LINC01152 was significantly upregulated in HBV-positive HCC tissues and cells treated by HBV X protein (HBx). Overexpression of LINC01152 could increase HCC cell proliferation and promote tumor formation in nude mice by directly binding to the promoter region of interleukin- (IL-) 23 to increase its transcription [6]. Hu et al. identified that lncRNA WEE2-AS1 was overexpressed in HCC tissues and was positively correlated to HBV infection. Compared with the low expression and HBV-negative groups, HBV-HCC patients with high expression of WEE2-AS1 exhibited the worst prognosis. WEE2-AS1 accelerated the proliferation, migration, invasion, and cell cycle progression of HCC cells by upregulating the downstream Fermitin family member 3 (FERMT3) [7]. Lin et al. observed that the expression of miR143HG was markedly decreased in HCC tissues and its expression was associated with the presence of HBV infection. Highly expressed miR143HG can serve as an independent prognostic factor to predict a good prognosis. *In vitro* analysis revealed that miR143HG may exert its tumor suppressor roles by sponging miR-155 and then promoting the expression of miR-155 target gene adenomatous polyposis coli (APC) which can inhibit the Wnt/*β*-catenin signaling pathway [8]. Fan et al. demonstrated that the expression of lncRNA n335586 was significantly increased in HBV-positive HCC tissues and cells. lncRNA n335586 may contribute to the migration, invasion, and metastasis of HCC cells by facilitating the expression of its host gene creatine kinase, mitochondrial 1A (CKMT1A), through competitively binding to miR-924 [9]. These findings suggest that lncRNAs may be potential therapeutic targets and prognostic biomarkers for HBV-HCC patients. However, the lncRNAs specifically associated with HBV-HCC remain rarely reported and most of these studies focused on the prognostic roles of single lncRNA, and the attempt to identify a lncRNA prognostic signature for HBV-HCC was limited [10].

The present study is aimed at further identifying a lncRNA prognostic signature for HBV-HCC patients through using the sequencing data in The Cancer Genome Atlas (TCGA) database and a microarray dataset deposited in the European Bioinformatics Institute (EMBL-EBI) which had the larger sample size compared with the dataset used by Liu et al. [10] (44 vs. 19). Moreover, the potential functions of lncRNAs were predicted by constructing a ceRNA regulatory network, in addition to a coexpression network which was used in the study of Liu et al. [10]. Hereby, our study may provide a novel lncRNA prognostic signature and identify potential therapeutic targets for HBV-HCC patients.

## 2. Materials and Methods

### 2.1. Data Source

The mRNA- and miRNA-sequencing data (level 3, Illumina HiSeq 2000 RNA Sequencing platform) of HCC were obtained from TCGA database portal (https://portal.gdc.cancer.gov/) on March 25, 2019, which included 373 HCC samples and 50 normal liver controls. A total of 95 HCC samples were used in this analysis because they (1) sequenced for both mRNAs and miRNAs, (2) belonged to HBV alone-positive samples, and (3) had complete clinical data (including survival). This dataset was used as the training set.

Furthermore, a microarray dataset was also collected from the EMBL-EBI database (https://www.ebi.ac.uk/arrayexpress/) under accession number E-TABM-36 (platform: GPL 96[HG-U133A] Affymetrix GeneChip Human Genome HG-U133A) [11] to serve as the validation set. This dataset included 65 samples, 44 of which were HBV-positive and had survival information.

### 2.2. Differential Expression Analysis

The lncRNAs and mRNAs in RNA-sequencing data were annotated based on the HUGO Gene Nomenclature Committee (HGNC; http://www.genenames.org/) [12]. The Linear Models for Microarray Data (LIMMA) method (version 3.34.7; https://bioconductor.org/packages/release/bioc/html/limma.html) [13] in the Bioconductor R package (version 3.4.1; http://www.R-project.org/) was applied for the identification of differentially expressed genes (DEGs), lncRNAs (DELs), and miRNAs (DEMs) which were defined as the false discovery rate (FDR) < 0.05 and ∣log_2_FC(fold change)∣ > 1. Bidirectional hierarchical clustering was performed using the pheatmap R package (version: 1.0.8; https://cran.r-project.org/web/packages/pheatmap) based on centered Pearson's correlation to generate heat maps of differentially expressed RNAs.

### 2.3. Identification of a Prognostic lncRNA Signature Using the Training Set

The survival package (version 2.41-1; http://bioconductor.org/packages/survivalr/) was used for all statistical analyses. First, univariate Cox regression analysis was performed to mine DEGs, DELs, and DEMs that were associated with overall survival (OS) in the training set (log-rank *p* value < 0.05). Second, multivariate Cox regression analysis was conducted to further select independent prognostic lncRNAs, which were used to construct a lncRNA prognostic signature model according to the following formula:

(1) $$\begin{array}{c} \text{Risk score}=\sum \beta_{\text{lncRNA}}\times {\text{Exp}}_{\text{lncRNA}}, \end{array}$$

where Exp_lncRNA_ was the expression level of DELs and *β*_lncRNA_ was the regression coefficient for the DELs in multivariate Cox hazard model analysis.

Third, using the median risk score as the threshold, the HBV-HCC patients were divided into the low-risk and high-risk groups. The Kaplan-Meier (K-M) survival curves were used to calculate the differences in OS between the high- and low-risk groups. The receiver operating characteristic (ROC) curve and area under the curve (AUC) were also applied to assess the predictive performance of the prognostic risk score.

### 2.4. Validation of the Prognostic Power of the Risk Score Using the Validation Dataset

The risk score identified in the training dataset was further calculated for the validation dataset. Patients were also divided into the high-risk and low-risk groups according to the risk score. The K-M survival and ROC curves were analyzed.

### 2.5. The Prognostic Independence of the Risk Score

Univariate and multivariate Cox regression analyses were conducted to assess whether the prognostic performance of signature risk score was independent of other clinical characteristics (including age, gender, neoplasm histologic grade, pathologic TNM stage, vascular invasion, and recurrence) for patients with HBV-HCC.

### 2.6. Construction of lncRNA Coexpression and ceRNA Networks

The DIANA-LncBase (version 2.0; http://carolina.imis.athena-innovation.gr/diana_tools/web/index.php?r=lncbasev2/index-predicted) [14] database was used to predict the interactions between signature DELs and prognostic DEMs. The starBase database (version 2.0; http://starbase.sysu.edu.cn/) [15] was used to predict the interactions between prognostic DEMs and prognostic DEGs. The DEL-DEM and DEM-DEG interactors which had opposite expression trend were integrated to construct the ceRNA network, which was visualized using the Cytoscape software (version 3.6.1; http://www.cytoscape.org/) [16].

The coexpression network was constructed based on the correlation between signature DELs and DEGs which was calculated using the tcor.test function (https://stat.ethz.ch/R-manual/R-devel/library/stats/html/cor.test.html) in R to generate the Pearson correlation coefficients (PCC). Only the coexpression pairs with PCC > 0.4 were selected to draw the network using Cytoscape (version 3.4; http://www.cytoscape.org/) [16].

### 2.7. Function and Pathway Enrichment

Gene Ontology (GO) terms and Kyoto Encyclopedia of Genes and Genomes (KEGG) pathway enrichment analyses were performed using clusterProfiler (version 3.6.0; http://bioconductor.org/packages/release/bioc/html/clusterProfiler.html) to reveal the functions of DEGs in the lncRNA-related coexpression and ceRNA networks [17]. FDR < 0.05 was set as the cut-off value.

## 3. Results

### 3.1. Differential Expression Analysis

**Figure 1:**
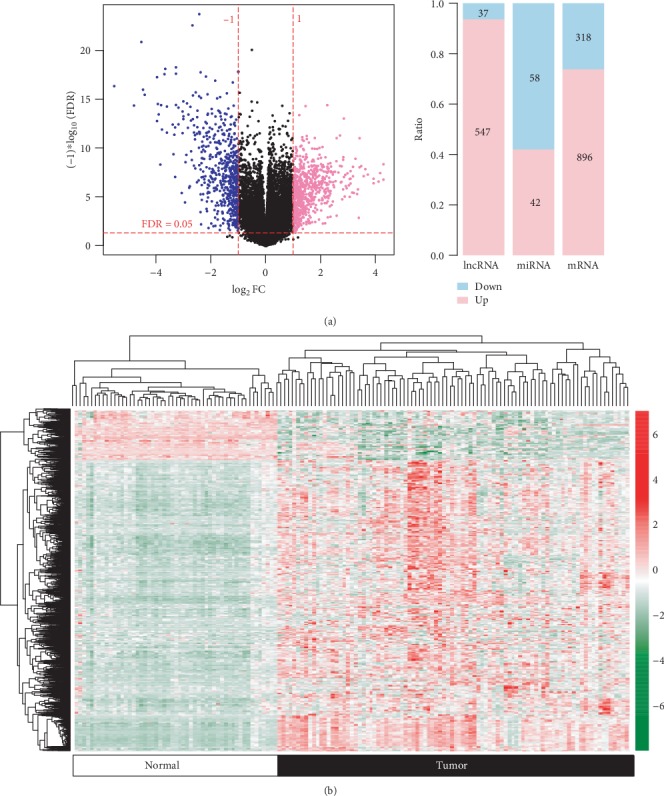
Differentially expressed RNAs. (a) Volcano plots were used to show the (left) distribution and (right) number of differentially expressed RNAs. Blue dot: downregulated RNAs; pink: upregulated RNAs; red horizontal dotted line: FDR < 0.05; red vertical dotted line: ∣log_2_FC∣ > 1. (b) Heat map of differentially expressed RNAs. Red: high expression; green: low expression. FDR: false discovery rate; FC: fold change.

After removing the expression value of zero, a total of 13,454 mRNAs, 1,238 lncRNAs, and 1,037 miRNAs were annotated. Using the LIMMA method, 1,898 differentially expressed RNAs were identified between the 95 HBV-HCC tissues and 50 controls, including 1,214 DEGs (318 downregulated; 896 upregulated), 584 DELs (37 downregulated; 547 upregulated), and 100 DEMs (42 downregulated; 58 upregulated) (Figure 1(a)). The heat map showed that the HBV-HCC and normal tissues were well distinguished by these differentially expressed RNAs (Figure 1(b)).

### 3.2. Identification of a lncRNA Signature with Prognostic Value

A total of 260 differentially expressed RNAs were identified to be significantly associated with OS by univariate Cox regression analysis, including 189 DEGs, 39 DELs, and 32 DEMs. To determine the optimal prognostic lncRNAs, multivariate Cox proportional hazards regression was further adopted to evaluate the prognostic independence of the above-identified 39 prognostic lncRNAs. As a result, 8 lncRNAs (TSPEAR-AS1, LINC00511, LINC01136, MKLN1-AS, LINC00506, KRTAP5-AS1, ZNF252P-AS1, and THUMPD3-AS1) were found to be still significant in the multivariate analysis (Table 1), indicating that these lncRNAs were independent prognostic biomarkers. As shown in Table 1, LINC00511, LINC01136, LINC00506, and THUMPD3-AS1 may be prognostic risk factors for OS because of negative coefficient and HR > 1 (that is, patients with high expression of lncRNAs had shorter survival) while TSPEAR-AS1, MKLN1-AS, KRTAP5-AS1, and ZNF252P-AS1 may be prognostic protective factors due to negative coefficient and HR < 1 (that is, patients with high expression of lncRNAs had longer survival).

**Figure 2:**
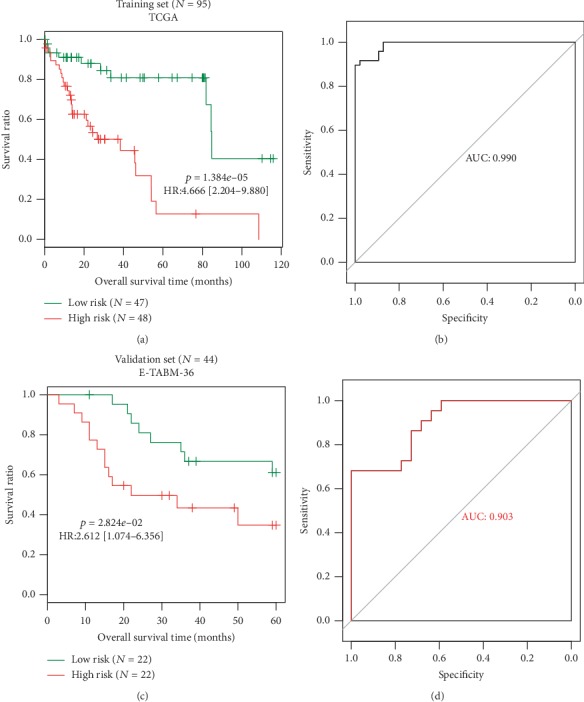
Prognosis prediction of the risk score model for overall survival in patients with HBV-HCC. (a) Kaplan-Meier plots of overall survival for patients with a high- or low-risk score of the training dataset. (b) ROC curves of the risk score based on 8-lncRNA signature using the training dataset. (c) Kaplan-Meier plots of overall survival for patients with a high- or low-risk score of the validation dataset. (d) ROC curves of the risk score based on an 8-lncRNA signature using the validation dataset. ROC: receiver operating characteristic; AUC: area under the ROC curve; HR: hazard ratio; TCGA: The Cancer Genome Atlas.

The risk score model of this eight-lncRNA signature was developed for each patient according to the following formula: risk score = (−3.188) × Exp_TSPEAR−AS1_ + (3.073) × Exp_LINC00511_ + (1.419) × Exp_LINC01136_ + (−5.167) × Exp_MKLN1−AS_ + (3.221) × Exp_LINC00506_ + (−7.167) × Exp_KRTAP5−AS1_ + (−11.93) × Exp_ZNF252P−AS1_ + (3.562) × Exp_THUMPD3−AS1_. The patients were classified into the high-risk and low-risk groups based on the median risk score. The K-M survival curve results showed that this eight-lncRNA signature can significantly differentiate the OS between the predicted two risk groups, with worse OS in HBV-HCC patients with high-risk scores (training dataset: HR = 4.666, 95%CI = 2.204‐9.880, Figure 2(a); validation dataset: HR = 2.612, 95%CI = 1.074‐6.356, Figure 2(c)). ROC analysis was subsequently performed to evaluate the prediction accuracy of this risk score of the eight-lncRNA signature. The results showed that the AUC of the ROC curve was, respectively, 0.990 and 0.903 for the training (Figure 2(b)) and validation datasets (Figure 2(d)), suggesting a good prediction performance.

### 3.3. Independence of the Eight-lncRNA Signature for Survival Prediction

Univariate and multivariate Cox regression analyses were also performed to further assess whether the prognostic value of the eight-lncRNA signature was independent of other clinical variables. As expected, the risk score status was identified as a significant prognostic factor in both of the univariate and multivariate Cox regression analyses (Table 2), indicating the independence of the eight-lncRNA signature for prognostic prediction.

### 3.4. Functional Analysis of lncRNAs

**Figure 3:**
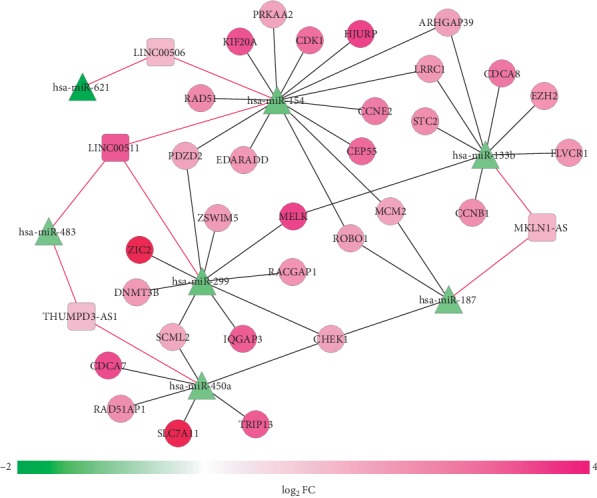
A competing endogenous RNA network among DELs, DEGs, and DEMs. Red: upregulated; green: downregulated. Circular: protein-coding genes; square: lncRNAs; triangle: miRNAs. DEGs: differentially expressed genes; DEMs: differentially expressed miRNAs; DELs: differentially expressed long noncoding RNAs; FC: fold change.

A total of 7 downregulated prognosis-related DEMs were predicted to interact with 4 upregulated signature DELs (LINC00511, LINC00506, MKLN1-AS, and THUMPD3-AS1) in the DIANA-LncBase database, which constituted 9 interaction pairs, while 30 upregulated prognostic DEGs were predicted to interact with 5 downregulated prognostic DEMs (hsa-miR-154, hsa-miR-187, hsa-miR-450a, hsa-miR-299, and hsa-miR-133b) by the starBase database, which constitute5d 39 interaction pairs. These interaction pairs were integrated to construct a lncRNA ceRNA network (Figure 3), in which THUMPD3-AS1-related ceRNA axes, such as THUMPD3-AS1-hsa-miR-450a-TRIP13 (thyroid hormone receptor interactor 13), may be potentially credible because the expression and prognosis trend was similar between this lncRNA and mRNA, but opposite between this lncRNA and miRNA (Table 1).

**Figure 4:**
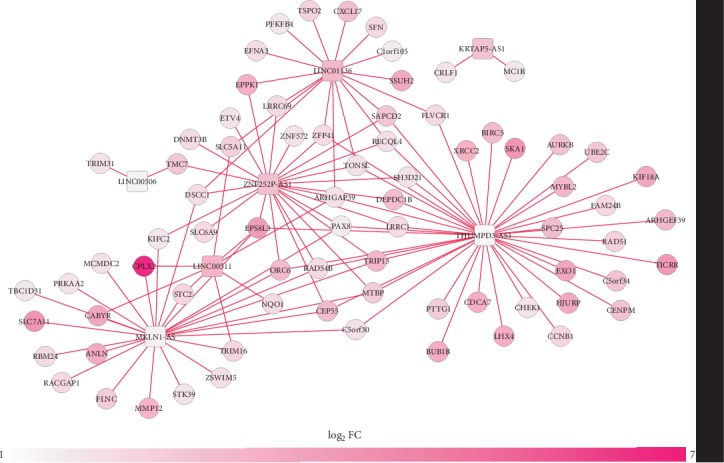
A coexpression network between DELs and DEGs. Red: upregulated; green: downregulated. Circular: protein-coding genes; square: lncRNAs. DEGs: differentially expressed genes; DELs: differentially expressed long noncoding RNAs; FC: fold change.

Furthermore, 7 upregulated signature DELs (LINC00511, LINC01136, MKLN1-AS, LINC00506, KRTAP5-AS1, ZNF252P-AS1, and THUMPD3-AS1) were predicted to coexpress with 75 prognostic DEGs according to the threshold of PCC > 0.4, which constituted 117 interaction pairs, and were used to construct the coexpression network (Figure 4). According to the principle of consistent expression and prognosis trend, the following coexpression pairs were suggested to be important, including LINC00511-CABYR (calcium binding tyrosine phosphorylation regulated) (PCC = 0.53), LINC01136-SFN (stratifin) (PCC = 0.41), THUMPD3-AS1-TRIP13 (PCC = 0.48), and KRTAP5-AS1-MC1R (melanocortin 1 receptor) (PCC = 0.43) (Table 1; Figure 4). Furthermore, LINC00506-ANLN (anillin actin binding protein) (PCC = 0.26), TSPEAR-AS1 (PCC = 0.000549)/MKLN1-AS (PCC = 0.21)/ZNF252P-AS1 (PCC = 0.13)-MC1R (melanocortin 1 receptor) coexpression relationships also complied with the principle, although their PCC were less than 0.4.

**Figure 5:**
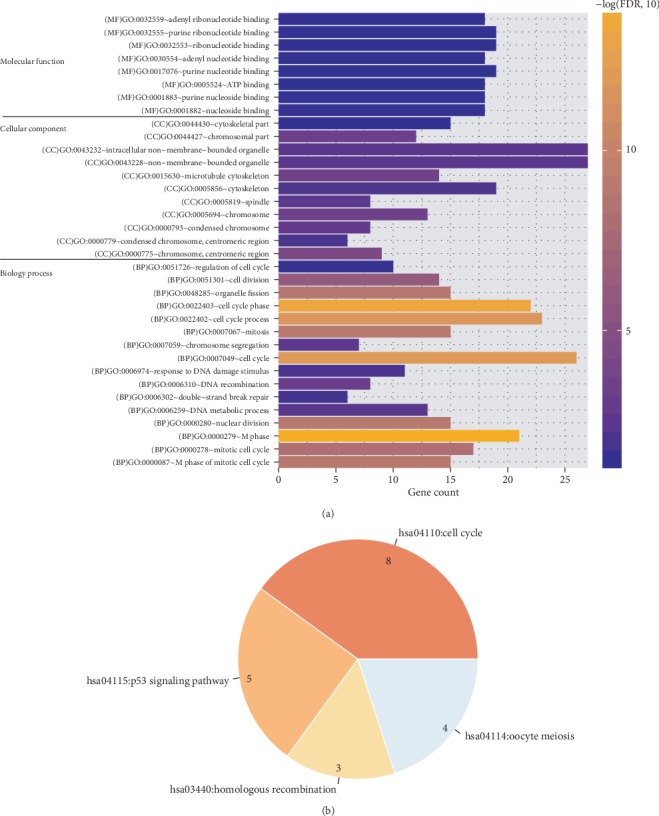
Function enrichment analysis. (a) Gene Ontology (GO). FDR: false discovery rate; BP: biology process; CC: cellular component; MF: molecular function. (b) Kyoto Encyclopedia of Genes and Genomes (KEGG) pathway.

Function enrichment analysis screened 35 GO terms and 4 KEGG pathways (Table 3; Figure 5) for the DEGs of the ceRNA and coexpression networks. All of the DEGs included in the above crucial ceRNA axes or coexpression pairs were enriched, including the GO:0000279~M phase (ANLN, MC1R, and TRIP13), GO:0022402~cell cycle process (ANLN, MC1R, and TRIP13), GO:0006974~response to DNA damage stimulus (SFN, TRIP13, and ANLN), GO:0005856~cytoskeleton (CABYR), hsa04110:cell cycle (SFN), and hsa04115:p53 signaling pathway (SFN).

## 4. Discussion

In the present study, an eight-lncRNA signature (TSPEAR-AS1, LINC00511, LINC01136, MKLN1-AS, LINC00506, KRTAP5-AS1, ZNF252P-AS1, and THUMPD3-AS1) was identified as an independent prognostic biomarker for HBV-positive HCC patients. ROC curve analysis showed that the risk score established by this eight-lncRNA signature had high prediction accuracy for OS, with AUC of 0.990 for the training set and 0.903 for the validation set, respectively. Further mechanism analyses uncovered upregulated THUMPD3-AS1 may function as a ceRNA to sponge hsa-miR-450a and then regulate TRIP13. In addition, THUMPD3-AS1 may also directly regulate TRIP13 by coexpression with it. The other lncRNAs may exert protective or risk roles for the development of HBV-positive HCC mainly by coexpression with their downstream genes, such as LINC00511-CABYR, LINC01136-SFN, LINC00506-ANLN, and KRTAP5-AS1/TSPEAR-AS1/MKLN1-AS/ZNF252P-AS1-MC1R. These genes were involved in cell cycle-related GO terms or KEGG pathways.

Although TCGA data with essentially the same samples were also used as the training set to screen the lncRNA signature in the study of Liu et al. [10], there were no common lncRNAs between our identified lncRNA signature and that of Liu et al. [10] (DGCR9, GBA3, HCG4, NAT8B, NBR2, PART1, RFPL1S, SLC22A18AS, and TCL6). This may be attributed to the analysis method difference (i.e., only lncRNAs in partial module were used for univariate Cox regression in Liu et al. [10], but all DELs in our study). Furthermore, only TSPEAR-AS1 (upregulated, protective) [18] and LINC00511 (upregulated, risk) [19], with the prognosis trend in line with ours, were reported as prognostic factors for the whole HCC, while KRTAP5-AS1 (upregulated, protective; similar to ours) [20] and THUMPD3-AS1 (upregulated, protective; contrast to ours) [21] were identified to be associated with OS in papillary thyroid carcinoma and lung adenocarcinoma, respectively. No studies investigated the prognostic values of LINC01136, MKLN1-AS, LINC00506, and ZNF252P-AS1. These findings indicated that our lncRNA signature may be a first-time identified biomarker of the prognosis for HBV-HCC.

Besides, the predictive accuracy of our eight-lncRNA signature was comparable with that of 9-lncRNA signature for HBV-HCC identified by Liu et al. (0.990 vs. 0.953) [10] and obviously higher than that of the published studies focusing on the whole HCC, such as Shi et al. (8-lncRNA signature, AUC = 0.769, 0.708, and 0.75 for training, testing, and entire set) [22], Yan et al. (7-lncRNA signature, AUC = 0.752) [23], Wang et al. (4-lncRNA signature, AUC = 0.73) [24], Ma et al. (4-lncRNA signature, AUC = 0.73) [25], Sui et al. (4-lncRNA signature, AUC = 0.709) [26], and Zhang et al. (10-lncRNA signature, AUC = 0.796) [27]. These results suggested that our eight-lncRNA signature may be a more effective, robust biomarker for HCC prognosis, particularly HBV-positive ones.

Several *in vitro* studies had demonstrated the functions of LINC00511 in cancer, but most of them focused on the ceRNA mechanism. The study of Zhang et al. suggested that highly expressed LINC0051 directly interacted with miR-29c to suppress its expression and then promoted the upregulation of CDK6, a direct target of miR-29c, leading to an increase in the cell viability of breast cancer cells [28]. Lu et al. concluded that LINC00511 promoted the proliferation, sphere-formation ability, and tumor growth in breast cancer cells by functioning as a ceRNA for miR-185-3p to positively recover E2F1 protein and promote the transcription of Nanog gene [29]. Furthermore, LINC00511-hsa-miR-29b-3p-VEGFA (vascular endothelial growth factor A) [30], LINC00511-miR-765-LAMC2 (laminin subunit gamma 2) [31], and LINC00511-miR-124-3p-CCND2 (cyclin D2) [32] ceRNA axes were also reported for pancreatic ductal adenocarcinoma, tongue squamous cell carcinoma, and glioma, respectively. But the coexpression mechanism of LINC00511 was rarely reported [33]. In this study, we speculated LINC00511 may coexpress with CABYR. Although no experiments were performed to confirm their relationships, previous studies on the roles of CABYR may indirectly explain our conclusion. For example, Li et al. found that the mRNA and protein levels of CABYR-c were significantly higher in HCC tissues than those in the adjacent noncancerous tissues. Silencing of CABYR-c by antisense oligonucleotides significantly inhibited the cell growth of HepG2 cells and arresting the cell cycle [34]. Similarly, knockdown of CABYR-a/b was also reported to increase the cell apoptosis and enhance the chemosensitivity [35]. In line with these studies, our study also identified that LINC00511 and CABYR were upregulated and predicted a poor prognosis.

THUMPD3-AS1 was not included in the ceRNA network of Li et al. [21], and thus, its potential function remained unknown. In this study, we predicted that upregulated THUMPD3-AS1 may directly coexpress with TRIP13 to promote its upregulated expression or regulate it by sponging hsa-miR-450a. Although no research was conducted to verify their interaction relationships, previous studies on the expression and roles of these miRNA and mRNA may indirectly prove our speculation. Ju et al. detected that the expression of TRIP13 was upregulated in HCC tissues and cell lines. Patients with higher expression levels of TRIP13 had significantly shorter survival periods [36]. Downregulation of TRIP13 inhibited HCC cell proliferation, migration, and invasion, promoted apoptosis and cell cycle arrest at S-phase *in vitro* [36], and suppressed the formation of tumor *in vivo* [37]. The study of Weng et al. revealed that miR-450a was significantly downregulated in HCC tissues and cells. Ectopic expression of miR-450a in HepG2 cells caused an inhibition of cell proliferation, the mechanism of which was related to downregulation of DNA methyltransferase 3a [38].

LINC01136 was a first-time identified oncogene in our study, and its role was unknown. We predicted it may exert tumor-promoting roles by coexpressing with SFN which had been demonstrated to be oncogenic previously as follows: SFN protein was previously identified to be upregulated in HCC via proteomics analyses [39]. SFN guided cell migration and invasion of breast cancer cells by stabilizing a complex of soluble actin [40]. Suppression of SFN expression by siRNA significantly reduced proliferation activity and the S-phase subpopulation of human lung adenocarcinoma cells and blocked tumor development in mice [41]. By analysis of ten datasets, Hu et al. found that high expression of SFN was significantly associated with worse OS in patients with ovarian cancer [42]. In agreement with these studies, we also found that SFN was upregulated and predicted a poor prognosis in HBV-HCC.

The function of LINC00506 was also not reported previously. We predicted it coexpressed with ANLN to promote carcinogenesis. ANLN was detected to be highly expressed in HCC tissues and cells infected with HBV. Higher ANLN expression was associated with a worse prognosis. Moreover, inhibition of ANLN resulted in growth restraint, colony formation reduction, and apoptosis activation [43] as well as survival time increase [44]. In accordance with these studies, we also found ANLN was highly expressed in HBV-HCC tissues and related to a poor prognosis.

The functions of TSPEAR-AS1 and KRTAP5-AS1 had been predicted based on the ceRNA network previously. For example, TSPEAR-AS1 may sponge miR-424 to regulate the expression of G protein subunit alpha L (GNAL) [18] and lncRNA KRTAP5-AS1 could act as a ceRNA to affect the functions of Claudin-4 [45]. However, their roles remain unclear. In this study, our study suggested that TSPEAR-AS1 and KRTAP5-AS1 as well as MKLN1-AS/ZNF252P-AS1 may exert protective roles for HBV-HCC by coexpressing with MC1R. The studies on MC1R mainly focused on melanoma, and the results indicated that patients had significantly longer OS in melanoma tumors with lower expression of MC1R [46], which was opposite with our expectation. Thus, further studies on MC1R in other cancers especially HBV-HCC are necessary.

There were some limitations in this study. First, the sample size was small. Therefore, large HBV-HCC cohorts should be collected to confirm the prognosis values of our lncRNA signature. Second, this study was only preliminary to predict the functions of lncRNAs in HBV-HCC and further wet experiments should be performed to verify their ceRNA (luciferase assay, overexpression or silencing) or coexpression (coimmunoprecipitation) mechanisms. Third, previous studies have demonstrated that the gene profiles induced by HBV, HCV, and other etiologies were different [47–49]. Thus, theoretically, a distinct signature or results using the same signature may be obtained when a similar analysis was performed [50], which should be confirmed in subsequent studies. Fourth, due to the limited description of treatment regimens in TCGA data, we could not investigate the influence of therapies (hepatectomy, radiofrequency ablation, transarterial embolization, and others) on the prognosis of HBV-HCC patients.

## 5. Conclusion

In the present study, we constructed an 8-lncRNA risk score model for survival prediction of HBV-HCC based on RNA-Seq and microarray datasets, with the prediction accuracy more than 90%. These signature lncRNAs may be involved in HBV-HCC by coexpressing with mRNAs (LINC00511-CABYR, THUMPD3-AS1-TRIP13, LINC01136-SFN, LINC00506-ANLN, and KRTAP5-AS1/TSPEAR-AS1/MKLN1-AS/ZNF252P-AS1-MC1R) or functioning as a ceRNA (THUMPD3-AS1-hsa-miR-450a-TRIP13) to regulate the cell cycle-related biological processes or pathways.

## Figures and Tables

**Table 1:** Prognostic RNAs significantly associated with OS.

	Symbol	logFC	FDR	*β*	HR	95% CI	*p* value	Type
lncRNA	TSPEAR-AS1	2.08	8.82*E*-14	-3.19	0.41	0.07-0.60	6.92*E*-04	Protective
	LINC00511	3.14	1.80*E*-10	3.07	2.16	1.26-4.78	4.31*E*-03	Risk
	LINC01136	3.05	2.18*E*-10	1.42	1.45	1.05-2.61	7.30*E*-03	Risk
	MKLN1-AS	1.39	1.27*E*-16	-5.17	0.17	0.10-0.34	1.33*E*-02	Protective
	LINC00506	1.32	1.90*E*-03	3.22	2.71	1.90-4.25	1.74*E*-02	Risk
	KRTAP5-AS1	2.67	7.82*E*-08	-7.17	0.08	0.02-0.37	2.26*E*-02	Protective
	ZNF252P-AS1	2.75	1.26*E*-16	-11.93	0.07	0.01-0.56	3.93*E*-02	Protective
	THUMPD3-AS1	1.24	6.12*E*-26	3.56	3.52	1.07-5.12	4.59*E*-02	Risk

miRNA	hsa-miR-450a	-1.14	8.15*E*-21	-0.29	0.75		0.15	Protective

mRNA	TRIP13	3.07	2.38*E*-20	0.71	2.04		1.40*E*-03	Risk
	MC1R	1.61	5.01*E*-09	-1.7	0.18		3.40*E*-02	Protective
	CABYR	3.50	1.42*E*-07	0.31	1.37		4.00*E*-03	Risk
	SFN	2.07	9.60*E*-14	0.28	1.32		5.60*E*-04	Risk
	ANLN	3.37	1.97*E*-22	0.39	1.48		3.40*E*-02	Risk

Multivariate analysis results for the lncRNAs and univariate analysis results for the miRNA and mRNAs. FC: fold change; FDR: false discovery rate; HR: hazard ratio; CI: confidence interval; OS: overall survival.

**Table 2:** Univariate and multivariate analyses of the lncRNA signature and survival.

Features	TCGA (*N* = 95)	Univariate cox		Multivariate cox	
		HR (95% CI)	*p* value	HR (95% CI)	*p* value
Age (mean ± SD, years)	61.21 ± 14.90	1.039 (1.008-1.070)	**1.26** **E** **-02**	1.004 (0.976-1.034)	7.66*E*-01
Gender (male/female)	58/37	1.296 (0.661-2.544)	4.49*E*-01	—	—
Neoplasm histologic grade (G1/G2/G3)	12/47/36	1.071 (0.646-1.775)	7.91*E*-01	—	—
Pathologic M (M0/M1/-)	60/2/33	1.088 (0.919-10.28)	6.33*E*-01	—	—
Pathologic N (N0/N1/-)	59/2/34	1.178 (0.849-16.23)	1.47*E*-01	—	—
Pathologic T (T1/T2/T3/T4/-)	38/33/21/2/1	1.131 (0.752-1.700)	5.55*E*-01	—	—
Pathologic stage (stage I/II/III/IV)	36/30/22/2	1.037 (0.697-1.543)	8.57*E*-01	—	—
Vascular invasion (yes/no/-)	38/47/10	2.421 (1.232-4.756)	**8.12** **E** **-03**	2.113 (1.013-4.408)	**4.63** **E** **-02**
Tumor recurrence (yes/no)	38/57	2.321 (1.217-4.429)	**8.56** **E** **-03**	1.664 (1.213-3.405)	**4.16** **E** **-02**
Risk score status (high/low)	48/47	4.666 (2.204-9.880)	**1.38** **E** **-05**	3.198 (1.424-7.184)	**4.88** **E** **-03**
Death (dead/alive)	38/57	—	—	—	—
Overall survival time (mean ± SD, months)	32.25 ± 29.77	—	—	—	—

SD: standard deviation; TCGA: The Cancer Genome Atlas; HR: hazard ratio; CI: confidence interval. Bold indicates the results with statistical significance (*p* < 0.05).

**Table 3:** Function enrichment for genes regulated by lncRNAs.

Category	Term	FDR	Genes
Biology process	GO:0000279~M phase	1.06*E*-14	EXO1, CDK1, XRCC2, KIF18A, CHEK1, ANLN, BIRC5, AURKB, PTTG1, CEP55, UBE2C, RAD51, CCNB1, SPC25, CDCA8, MC1R, BUB1B, RAD54B, SKA1, DSCC1, TRIP13
	GO:0022403~cell cycle phase	5.37*E*-14	EXO1, CDK1, XRCC2, KIF18A, CHEK1, ANLN, BIRC5, AURKB, PTTG1, CEP55, UBE2C, RAD51, CCNB1, SPC25, CDCA8, MC1R, MTBP, BUB1B, RAD54B, SKA1, DSCC1, TRIP13
	GO:0007049~cell cycle	1.17*E*-12	XRCC2, CHEK1, ANLN, CEP55, AURKB, PTTG1, CCNE2, SPC25, CDCA8, MC1R, HJURP, MTBP, SKA1, TRIP13, EXO1, CDK1, KIF18A, BIRC5, MCM2, UBE2C, RACGAP1, RAD51, CCNB1, BUB1B, RAD54B, DSCC1
	GO:0022402~cell cycle process	2.15*E*-12	EXO1, CDK1, XRCC2, KIF18A, CHEK1, ANLN, BIRC5, AURKB, PTTG1, CEP55, RACGAP1, UBE2C, RAD51, CCNB1, SPC25, CDCA8, MC1R, MTBP, BUB1B, RAD54B, SKA1, DSCC1, TRIP13
	GO:0000280~nuclear division	1.02*E*-09	CDK1, KIF18A, ANLN, BIRC5, AURKB, PTTG1, CEP55, UBE2C, CCNB1, SPC25, CDCA8, MC1R, BUB1B, SKA1, DSCC1
	GO:0007067~mitosis	1.02*E*-09	CDK1, KIF18A, ANLN, BIRC5, AURKB, PTTG1, CEP55, UBE2C, CCNB1, SPC25, CDCA8, MC1R, BUB1B, SKA1, DSCC1
	GO:0000087~M phase of mitotic cell cycle	1.31*E*-09	CDK1, KIF18A, ANLN, BIRC5, AURKB, PTTG1, CEP55, UBE2C, CCNB1, SPC25, CDCA8, MC1R, BUB1B, SKA1, DSCC1
	GO:0048285~organelle fission	1.77*E*-09	CDK1, KIF18A, ANLN, BIRC5, AURKB, PTTG1, CEP55, UBE2C, CCNB1, SPC25, CDCA8, MC1R, BUB1B, SKA1, DSCC1
	GO:0000278~mitotic cell cycle	7.02*E*-09	CDK1, KIF18A, CHEK1, ANLN, BIRC5, AURKB, PTTG1, CEP55, UBE2C, CCNB1, SPC25, CDCA8, MC1R, MTBP, BUB1B, SKA1, DSCC1
	GO:0051301~cell division	7.44*E*-07	CDK1, ANLN, BIRC5, AURKB, PTTG1, CEP55, UBE2C, RACGAP1, CCNB1, CCNE2, SPC25, CDCA8, BUB1B, SKA1
	GO:0006310~DNA recombination	6.41*E*-04	EXO1, RECQL4, XRCC2, RAD51AP1, CHEK1, RAD54B, TRIP13, RAD51
	GO:0007059~chromosome segregation	2.44*E*-03	SPC25, HJURP, KIF18A, BIRC5, SKA1, PTTG1, DSCC1
	GO:0006259~DNA metabolic process	3.22*E*-03	EXO1, RECQL4, XRCC2, RAD51AP1, CHEK1, PTTG1, MCM2, RAD51, CCNE2, RAD54B, DNMT3B, TRIP13, DSCC1
	GO:0006974~response to DNA damage stimulus	8.66*E*-03	EXO1, RECQL4, CDK1, XRCC2, RAD51AP1, CHEK1, RAD54B, SFN, PTTG1, TRIP13, RAD51
	GO:0006302~double-strand break repair	1.25*E*-02	RECQL4, XRCC2, RAD51AP1, RAD54B, TRIP13, RAD51
	GO:0051726~regulation of cell cycle	2.38*E*-02	CCNE2, CCNB1, CDK1, MTBP, BUB1B, BIRC5, CHEK1, ANLN, SFN, UBE2C

Cellular component	GO:0000775~chromosome, centromeric region	4.42*E*-05	SPC25, CDCA8, CENPM, HJURP, BUB1B, BIRC5, SKA1, AURKB, DSCC1
	GO:0015630~microtubule cytoskeleton	2.92*E*-04	KIFC2, CDK1, KIF18A, BIRC5, CHEK1, CEP55, AURKB, RACGAP1, CCNB1, CDCA8, MC1R, BUB1B, SKA1, KIF20A
	GO:0005694~chromosome	3.28*E*-04	CENPM, CHEK1, BIRC5, AURKB, MCM2, RAD51, SPC25, CDCA8, HJURP, BUB1B, SKA1, DNMT3B, DSCC1
	GO:0044427~chromosomal part	4.63*E*-04	SPC25, CDCA8, CENPM, HJURP, BUB1B, BIRC5, CHEK1, SKA1, MCM2, AURKB, DNMT3B, DSCC1
	GO:0000793~condensed chromosome	1.09*E*-03	SPC25, CENPM, HJURP, BUB1B, CHEK1, SKA1, AURKB, RAD51
	GO:0005819~spindle	2.63*E*-03	CDK1, CDCA8, KIF18A, BUB1B, BIRC5, SKA1, AURKB, RACGAP1
	GO:0043232~intracellular non-membrane-bounded organelle	3.08*E*-03	KIFC2, CHEK1, ANLN, AURKB, CEP55, SPC25, CDCA8, MC1R, HJURP, STK39, SKA1, DNMT3B, ETV4, CDK1, CENPM, EPPK1, KIF18A, BIRC5, MCM2, FLNC, RACGAP1, CABYR, RAD51, CCNB1, BUB1B, DSCC1, KIF20A
	GO:0043228~non-membrane-bounded organelle	3.08*E*-03	KIFC2, CHEK1, ANLN, AURKB, CEP55, SPC25, CDCA8, MC1R, HJURP, STK39, SKA1, DNMT3B, ETV4, CDK1, CENPM, EPPK1, KIF18A, BIRC5, MCM2, FLNC, RACGAP1, CABYR, RAD51, CCNB1, BUB1B, DSCC1, KIF20A
	GO:0005856~cytoskeleton	6.60*E*-03	KIFC2, CDK1, EPPK1, KIF18A, CHEK1, ANLN, BIRC5, AURKB, CEP55, FLNC, RACGAP1, CABYR, CCNB1, CDCA8, MC1R, BUB1B, STK39, SKA1, KIF20A
	GO:0000779~condensed chromosome, centromeric region	8.57*E*-03	SPC25, CENPM, HJURP, BUB1B, SKA1, AURKB
	GO:0044430~cytoskeletal part	2.60*E*-02	KIFC2, CDK1, KIF18A, CHEK1, ANLN, BIRC5, CEP55, AURKB, RACGAP1, CCNB1, CDCA8, MC1R, BUB1B, SKA1, KIF20A

Molecular function	GO:0001882~nucleoside binding	2.49*E*-02	KIFC2, RECQL4, CDK1, XRCC2, PFKFB4, KIF18A, CHEK1, MCM2, AURKB, UBE2C, RAD51, BUB1B, STK39, RAD54B, PRKAA2, MELK, KIF20A, TRIP13
	GO:0032559~adenyl ribonucleotide binding	2.59*E*-02	KIFC2, RECQL4, CDK1, XRCC2, PFKFB4, KIF18A, CHEK1, MCM2, AURKB, UBE2C, RAD51, BUB1B, STK39, RAD54B, PRKAA2, MELK, KIF20A, TRIP13
	GO:0001883~purine nucleoside binding	2.87*E*-02	KIFC2, RECQL4, CDK1, XRCC2, PFKFB4, KIF18A, CHEK1, MCM2, AURKB, UBE2C, RAD51, BUB1B, STK39, RAD54B, PRKAA2, MELK, KIF20A, TRIP13
	GO:0030554~adenyl nucleotide binding	3.20*E*-02	KIFC2, RECQL4, CDK1, XRCC2, PFKFB4, KIF18A, CHEK1, MCM2, AURKB, UBE2C, RAD51, BUB1B, STK39, RAD54B, PRKAA2, MELK, KIF20A, TRIP13
	GO:0032555~purine ribonucleotide binding	3.27*E*-02	KIFC2, RECQL4, CDK1, XRCC2, PFKFB4, KIF18A, CHEK1, MCM2, AURKB, UBE2C, RAD51, MC1R, BUB1B, STK39, RAD54B, PRKAA2, MELK, KIF20A, TRIP13
	GO:0032553~ribonucleotide binding	3.27*E*-02	KIFC2, RECQL4, CDK1, XRCC2, PFKFB4, KIF18A, CHEK1, MCM2, AURKB, UBE2C, RAD51, MC1R, BUB1B, STK39, RAD54B, PRKAA2, MELK, KIF20A, TRIP13
	GO:0005524~ATP binding	4.36*E*-02	KIFC2, RECQL4, CDK1, XRCC2, PFKFB4, KIF18A, CHEK1, MCM2, AURKB, UBE2C, RAD51, BUB1B, STK39, RAD54B, PRKAA2, MELK, KIF20A, TRIP13
	GO:0017076~purine nucleotide binding	4.65*E*-02	KIFC2, RECQL4, CDK1, XRCC2, PFKFB4, KIF18A, CHEK1, MCM2, AURKB, UBE2C, RAD51, MC1R, BUB1B, STK39, RAD54B, PRKAA2, MELK, KIF20A, TRIP13

KEGG pathway	hsa04110:cell cycle	6.96*E*-04	CCNE2, CCNB1, CDK1, BUB1B, CHEK1, MCM2, SFN, PTTG1
	hsa04115:p53 signaling pathway	1.84*E*-02	CCNE2, CCNB1, CDK1, CHEK1, SFN
	hsa03440:homologous recombination	4.58*E*-02	XRCC2, RAD54B, RAD51
	hsa04114:oocyte meiosis	4.92*E*-02	CCNE2, CCNB1, CDK1, PTTG1

FDR: false discovery rate; GO: Gene Ontology; KEGG: Kyoto Encyclopedia of Genes and Genomes.

## Data Availability

The origin RNA-seq data used in our study were all downloaded from the TCGA (https://portal.gdc.cancer.gov/) and EMBL-EBI (https://www.ebi.ac.uk/arrayexpress/).
